# Targeting CD36-Mediated Lipid Metabolism by Selective Inhibitor-Augmented Antitumor Immune Responses in Oral Cancer

**DOI:** 10.3390/ijms25179438

**Published:** 2024-08-30

**Authors:** Mayu Takaichi, Hidetake Tachinami, Danki Takatsuka, Amirmoezz Yonesi, Kotaro Sakurai, Muhammad Irfan Rasul, Shuichi Imaue, Shin-Ichi Yamada, Muhammad Ruslin, Manabu Yamazaki, Jun-Ichi Tanuma, Makoto Noguchi, Kei Tomihara

**Affiliations:** 1 Department of Oral and Maxillofacial Surgery, Faculty of Medicine, Academic Assembly, University of Toyama, Toyama 930-0194, Japan; takaichi@med.u-toyama.ac.jp (M.T.); hidetake0506@gmail.com (H.T.); tdanki@med.u-toyama.ac.jp (D.T.); sakurai@med.u-toyama.ac.jp (K.S.); simaue@med.u-toyama.ac.jp (S.I.); shinshin@med.u-toyama.ac.jp (S.-I.Y.); toyama.mnoguchi@gmail.com (M.N.); 2 Department of Oral and Maxillofacial Surgery, Hasanuddin University, Makassar 90245, Indonesia; irfanrasul@unhas.ac.id (M.I.R.); mruslin@unhas.ac.id (M.R.); 3 Division of Oral Pathology, Faculty of Dentistry & Graduate School of Medical and Dental Sciences, Niigata University, Niigata 951-8514, Japan; manyamaz@dent.niigata-u.ac.jp (M.Y.); tanuma@dent.niigata-u.ac.jp (J.-I.T.); 4 Division of Oral and Maxillofacial Surgery, Faculty of Dentistry & Graduate School of Medical and Dental Sciences, Niigata University, Niigata 951-8514, Japan

**Keywords:** CD36, sulfosuccinimidyl oleate sodium, immunotherapy, oral squamous cell carcinoma

## Abstract

The fatty acid receptor CD36 is expressed on various malignant cells and is suggested to contribute to tumor progression. CD36 is also expressed by several immune cells and involved in immune responses and may be a potential target in cancer immunotherapy. In this study, we investigated whether the selective inhibition of CD36 can inhibit tumor progression and facilitate an antitumor immune response in oral squamous carcinoma cells (OSCCs). We assessed the effects of sulfosuccinimidyl oleate sodium (SSO), a CD36 inhibitor, on the proliferation apoptosis and alteration in tumor cell surface expression levels of immune accessory molecules in vitro. We also assessed whether SSO-treated OSCCs could promote a T cell response via a Mixed Lymphocyte Reaction (MLR) assay. We also investigated the direct antitumor effects and immunomodulatory effects of SSO using a mouse oral cancer OSCC model. SSO treatment significantly inhibited OSCC proliferation, increased apoptotic cell death, and upregulated the cell surface expression of several immune accessory molecules, including CD83, MHC-Class II, and PD-L1. SSO-treated OSCCs augmented T cell proliferation following MLR. In vivo SSO administration significantly attenuated mouse tumor growth with an increased proportion of immune cells, including CD4^+^ T, CD8^+^ T, and dendritic cells; it also decreased the proportion of immune suppressive cells, such as myeloid-derived suppressor and regulatory T cells. These results suggest that the selective inhibition of CD36 can induce direct and indirect antitumor effects by facilitating host antitumor immune responses in OSCCs.

## 1. Introduction

Oral squamous cell carcinoma (OSCC) is the most common malignancy of the head and neck, and the incidence of OSCC is predicted to increase by up to 40% worldwide by 2040 [1]. The major risk factors of OSCC include smoking, alcohol, human papillomavirus infection, and a particularly high incidence in Asian countries where chewing tobacco is common [2,3,4,5].

Although recent advances in treatment have significantly improved the patient outcomes of OSCC, it remains problematic for the strategy in cases of high-grade tumors that are refractory to conventional therapies. Therefore, novel treatment strategies are desired to be developed.

Cancer immunotherapy with immune checkpoint inhibitors (ICIs) has been clinically successful in many cancers, including OSCCs. Particularly, the potent antitumor effects of ICIs for recurrence and/or metastasis cancers are expected [6,7]. However, the clinical efficacies of ICIs are low and novel drugs in combination that improve the efficacy of ICIs are required.

The mechanism of tumor growth mediated by lipid metabolism has been elucidated in the tumor microenvironment (TME). Cancer cells proliferate by fatty acids (FAs) uptake via utilizing plasma membrane FA protein transporters, such as CD36, the family of FA transport proteins (SLC27), and plasma membrane FA-binding proteins (FABPs) [8]. These transporters have been shown to be highly expressed in cells of multiple cancer types [8].

Originally, it was well known that CD36 is homeostatically expressed in various types of cells (adipocytes, monocytes, macrophages, platelets, erythrocytes, dendritic cells (DCs), endothelial cells, cardiomyocytes, and epithelial cells) and involved in functions such as angiogenesis, cell adhesion, apoptosis, inflammatory mechanisms, and the lipid metabolism of these cells [9,10,11,12]. However, recent studies have also focused on the pro-tumor roles of CD36. Several studies have revealed its role in the proliferation or metastasis of multiple types of cancer cells, including breast cancer, hepatocellular carcinoma, colorectal cancer, ovarian cancer, bladder cancer, esophageal cancer, glioblastoma, and leukemia [13,14,15,16,17,18,19,20,21,22,23,24,25]. Our study and a study from another group also revealed that CD36 could promote the proliferation and migration of OSCCs [26,27].

Recently, the functional role of CD36 in tumor immunity has generated much interest, particularly in immune evasion in cancer. It has been demonstrated that CD36 contributed to the suppression in immune effector cells such as CD8^+^ T cells and CD4^+^ T cells [28,29]. Moreover, it has also been demonstrated that CD36 is expressed by immunosuppressive cells, such as regulatory T cells (Tregs) and myeloid-derived suppressor cells (MDSCs), and is associated with immunosuppression in cancer [30,31]. In dendritic cells (DCs), the overexpression of CD36 has also been reported to reduce the priming capacity of CD4^+^ T cells [32]. Therefore, as a therapeutic target in cancer immunotherapy, strategies targeting CD36 have garnered great interest [22,28,29,30]. However, little is known about the role of CD36 in the host immune responses in OSCC.

In the present study, we investigated whether the selective inhibition of CD36 could exert the direct antitumor effect and the immunomodulatory effects in OSCCs to elucidate the role of CD36-mediated lipid metabolism in OSCC tumor-bearing hosts. This is the first report to demonstrate the regulatory function of CD36 in the host immune responses in OSCCs.

## 2. Result

### 2.1. The Selective Inhibition of the CD36-Exerted Inhibitory Effect on the Proliferation of OSCCs, Accompanied by Increased Apoptotic Cell Death

**Figure 1:**
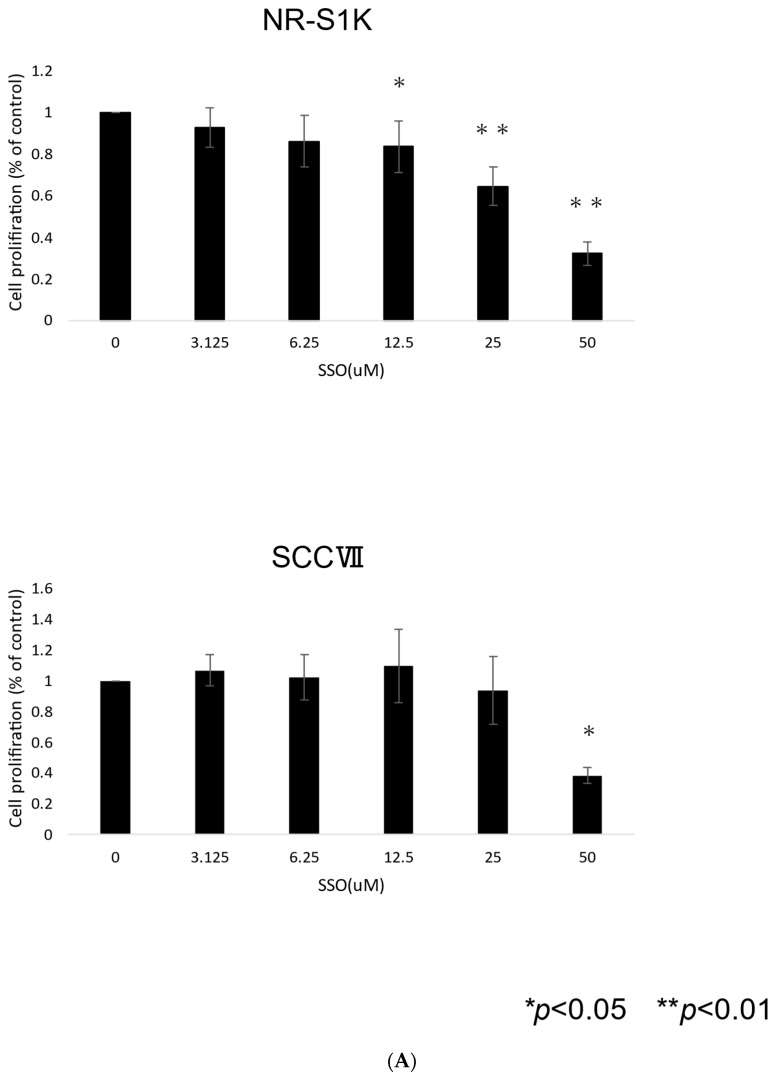

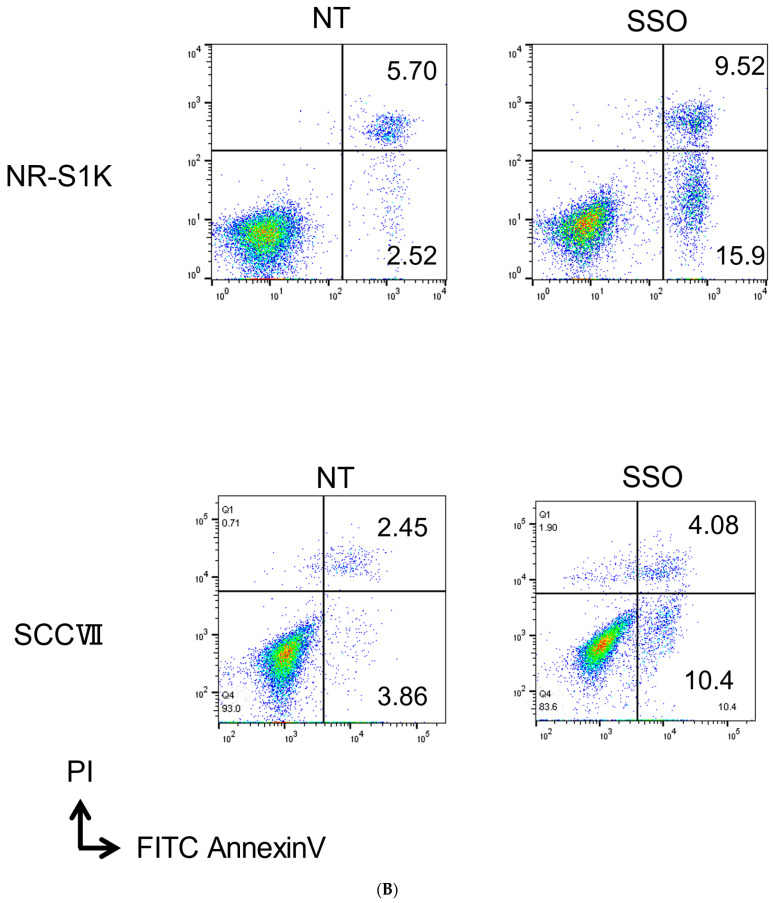
The growth inhibitory effect on OSCCs by SSO. (**A**) OSCCs were cultured in the presence or absence of various concentrations (3.125–50 μM) of SSO for 48 h, and the cell proliferation was measured by the WST-1 cell proliferation assay. The results are presented as mean ± standard deviation from quadruplet determinations. An asterisk indicates a significant difference between the two groups (*p* < 0.05). Experiments were performed three times and similar results were obtained. Representative data from one experiment are shown. (**B**) OSCCs were cultured in the presence or absence of SSO (50 μM) for 48 h, and then the cells were stained with propidium iodide (PI) and annexin V for quantification. Apoptotic cell death was determined by double staining with Annexin V and propidium iodide. The X-axis indicates Annexin V fluorescence and the Y-axis indicates propidium iodide fluorescence. Cells in the lower right and upper right quadrants represent early and late apoptotic cells. Experiments were performed in triplicate and similar results were obtained. Representative data from one experiment are shown.

To confirm the direct antitumor effect of CD36 inhibitors on OSCCs, we investigated the effects of SSO, a CD36 inhibitor, on the cell proliferation and apoptosis of NR-S1K cells and SCCVII cells using in vitro assays. As shown in Figure 1A, the viability of NR-S1K cells and SCCVII cells was significantly reduced after SSO treatment in a dose-dependent manner. 

Furthermore, SSO treatment also significantly increased the proportion of apoptotic cell death of NR-S1K cells and SCCVII cells (Figure 1B).

These results indicated that the selective inhibition of CD36 has a direct antitumor effect on OSCCs.

### 2.2. The Selective Inhibition of CD36-Altered Surface Antigen Expression in OSCCs

**Figure 2:**
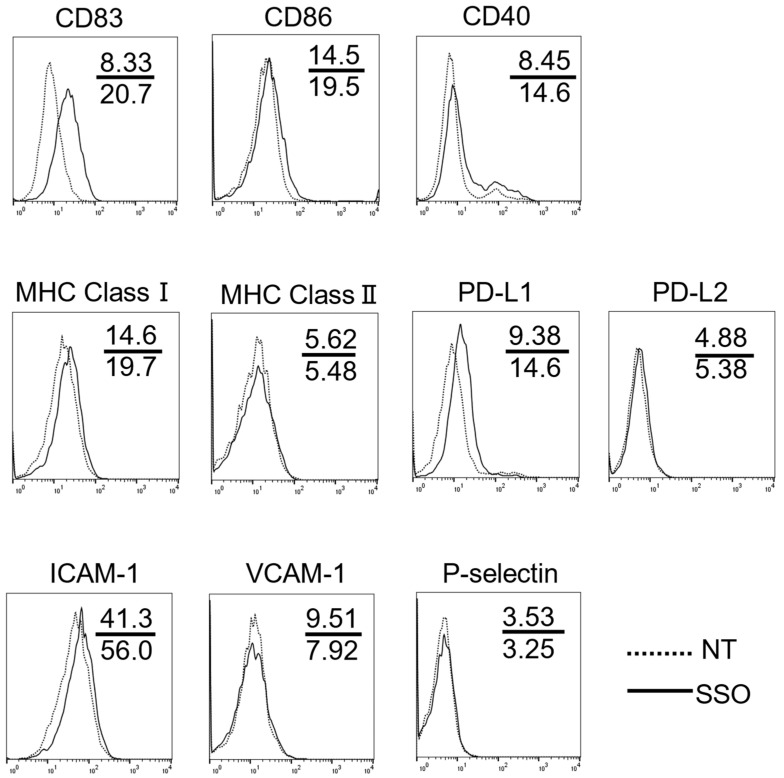
Phenotypic alteration of OSCCs by SSO. OSCCs were cultured in the presence or absence of 50 μM SSO for 48 h, and the cell surface expression of several immune accessary molecules and adhesion molecules was analyzed using flow cytometry. Experiments were performed in triplicate and similar results were obtained. Representative histograms from one experiment are shown. Dashed lines and solid lines indicate results for non-treated (NT) cells and SSO-treated cells. Numbers in each panel indicate the mean fluorescence intensity of each molecule in NT cells (upper) and SSO-treated cells (lower).

To confirm the effect of the selective inhibition of CD36 on the surface antigen expression changes of OSCCs, we compared the expression level of various T cell costimulatory and adhesion molecules on OSCCs with or without SSO treatment. As shown in Figure 2, the OSCCs treated with SSO showed increased levels of costimulatory molecules, including CD83, MHC class I, and PD-L1, compared to non-treated cells. Conversely, there were no changes in the expression levels of CD86, CD40, MHC-class II, orPD-L2 molecules. 

Furthermore, OSCCs treated with SSO showed increased levels of ICAM-1, but not VCAM-1 and P-selectin, compared to non-treated cells. 

OSCCs treated with SSO showed increased levels of CD36 compared to non-treated cells (Supplementary Figure S1). 

These results indicated that the selective inhibition of CD36 induces phenotype alterations of OSCCs, which may facilitate antitumor T cell responses.

### 2.3. The Selective Inhibition of CD36-Induced Phenotype Alteration of OSCCs That Facilitate T Cell Responses

**Figure 3:**
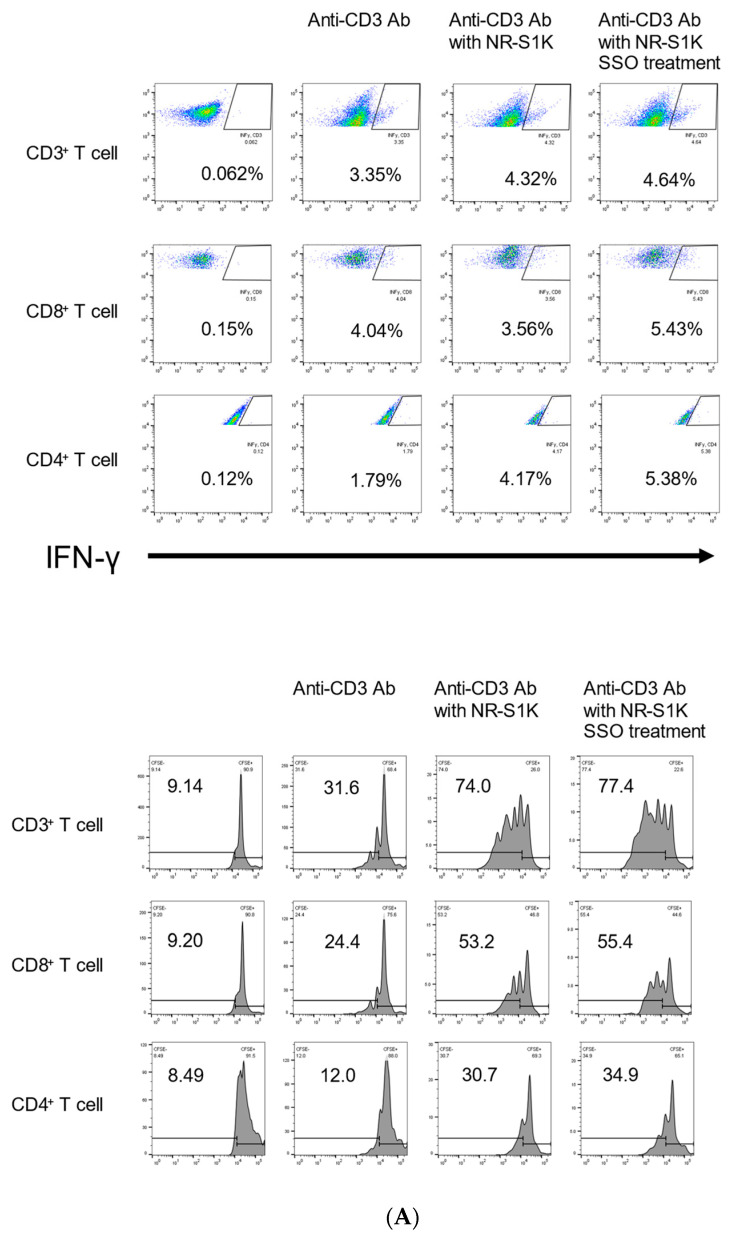

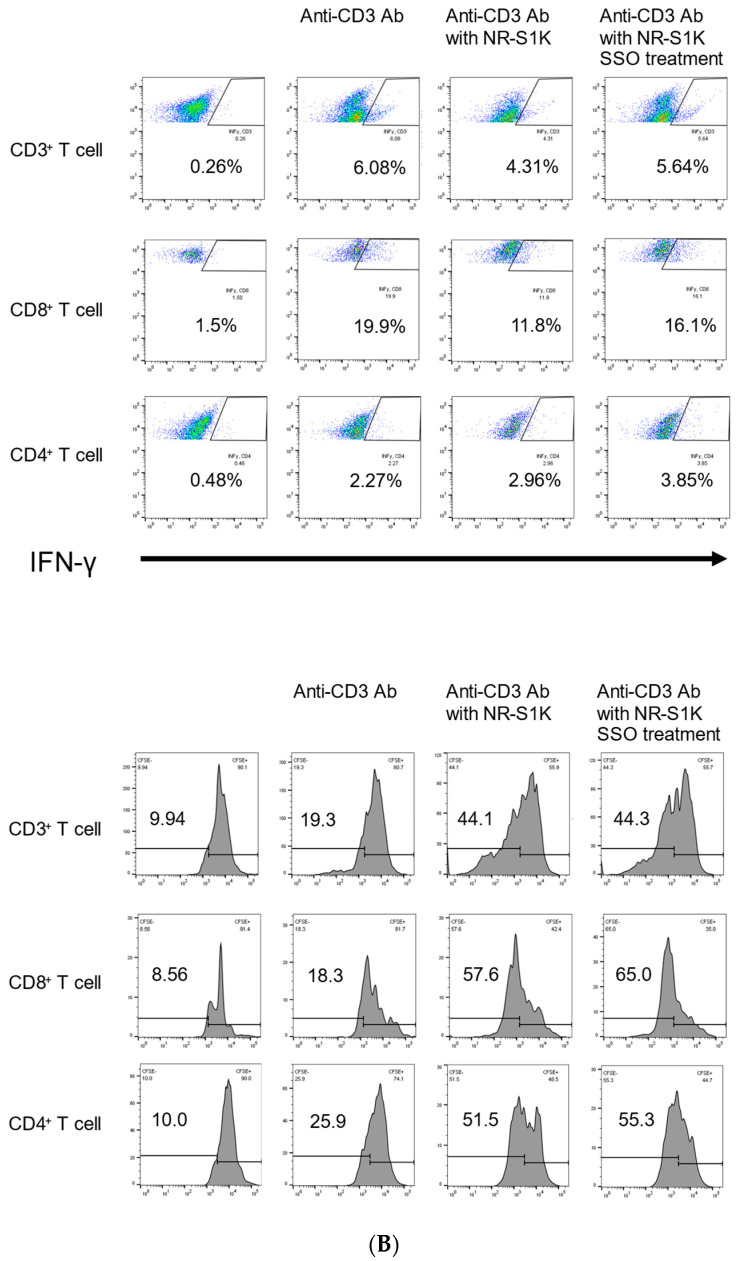
Augmented T cell responses by SSO-treated OSCCs. An in vitro Mixed Lymphocyte Reaction (MLR) assay was performed. NR-S1K cells were pretreated with 50μM SSO for 48 h. Then, lymph node cells (**A**) or spleen cells (**B**) from naive mice and NR-S1K cells (1 × 10^4^) with or without SSO pretreatment were cocultured in a 96-well U-bottom culture plate in the presence of 0.5 μg/mL anti-CD3 for 72 h. Cells were restimulated with 50 ng/mL PMA, 500 ng/mL ionomycin, and 4 μM monensin for 4 h before the end of the culture. The intracellular IFN-γ of T cells was determined using flow cytometry. Experiments were performed three times and similar results were obtained. Representative scatter plots from one experiment are shown. Furthermore, CFSE-labeled T cells from lymph node cells (**A**) or spleen cells (**B**) were cocultured with NR-S1K cells with or without SSO pretreatment. T cells were gated with the same gating as in the experiment for IFN-γ levels. T cell proliferation was measured by CFSE dilution. Experiments were performed in triplicate and similar results were obtained. Representative histograms from one experiment are shown. In both experiments, the control is the figure in the second column from the right.

To confirm changes in the T cell immune response for OSCCs with increased levels of costimulatory molecules due to the selective inhibition of CD36, we compared the capacity to stimulate T cells by a mixed-lymphocyte reaction (MLR) using OSCCs with or without SSO treatment [33]. As shown in Figure 3, IFN-γ-producing T cells were increased when cultured with the OSCCs that were treated with SSO compared with the OSCCs that were not treated. Furthermore, we compared T cell proliferation by coculturing with OSCC with or without SSO treatment. The CFSE-labeled T cells cocultured with the SSO-pretreated OSCCs had more proliferation than did the T cells cocultured with non-treated OSCCs (Figure 3). These results indicated that the selective inhibition of CD36 alters the phenotype of the OSCCs that facilitate T cell responses.

### 2.4. The Selective Inhibition of CD36-Exerted Antitumor Effects against an In Vivo Mouse Oral Cancer Model

**Figure 4:**
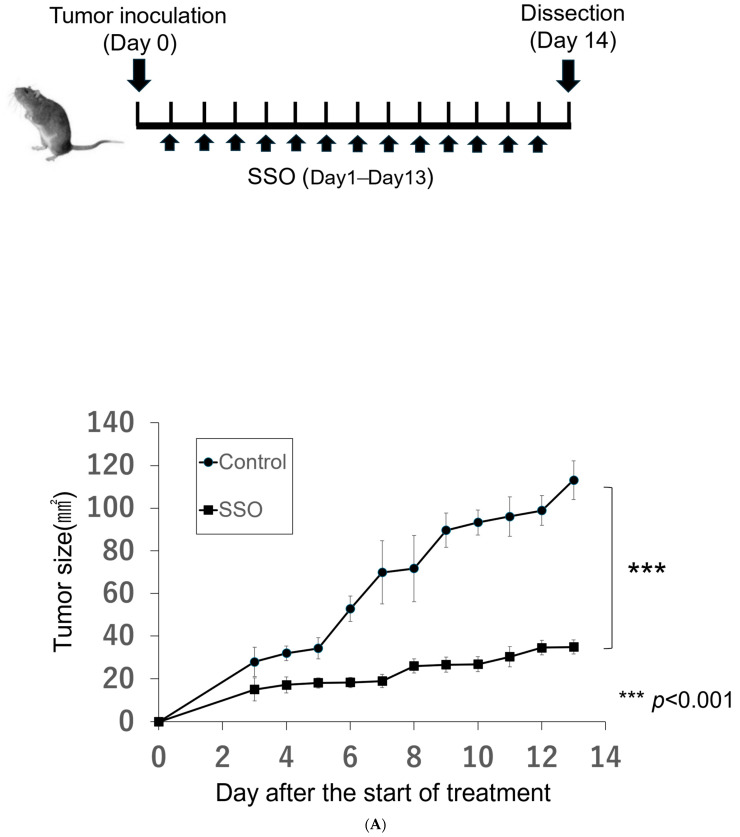

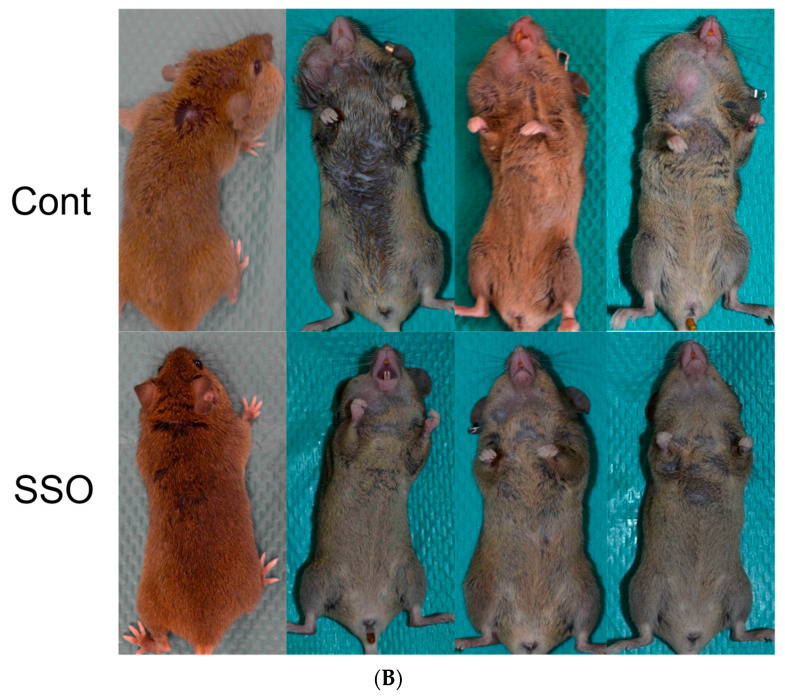
Growth inhibitory effect by SSO in a mouse model of OSCCs. Mice were challenged with NR-S1K cells. The mice were injected intraperitoneally with a dose of 20 mg/kg of SSO or saline every day from day 1 to day 13. (**A**) Tumor sizes in mice were measured at the indicated time points (*n* = 4/group); *** *p* < 0.01, NT vs. SSO. (**B**) Photos of tumor-bearing mice at the time of dissection. The upper images are of saline-treated control mice and the lower images are of SSO-treated mice.

To confirm the growth inhibitory effect on OSCCs via the CD36 inhibitor in vivo, the tumor growth was assessed during SSO treatment in a mouse model of OSCCs. As shown in Figure 4, SSO-treated OSCC mice showed significantly delayed tumor growth compared with the control mice. These results indicate that the SSO treatment in OSCC model mice may have been effective.

### 2.5. The In Vivo Administration of CD36 Inhibitor and the Modulated Distribution of Immune Cell Populations in a Mouse OSCC Model

To evaluate the immunological alterations on OSCCs via CD36 inhibitor in vivo, we compared immune cell populations between SSO-treated OSCC mice and control mice. Tumors, spleens, peripheral blood, peripheral lymph nodes, and cervical lymph nodes were harvested and the percentages of different immune cell types in each organ were determined after 14 days of SSO treatment. 

**Figure 5:**
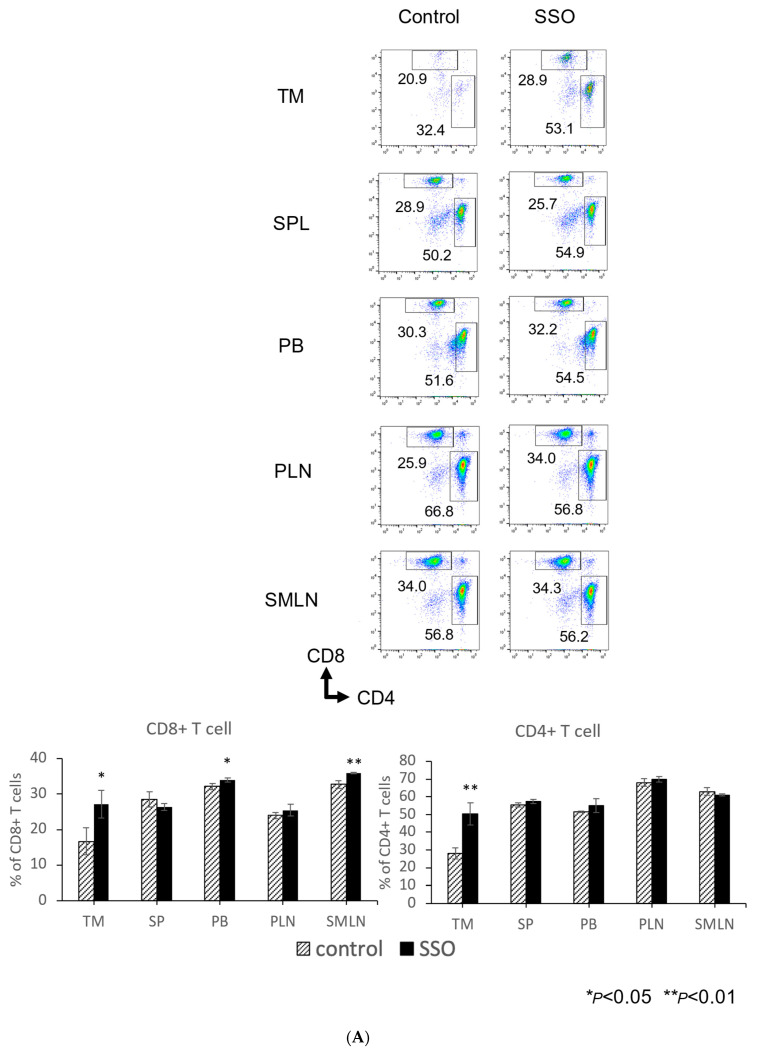

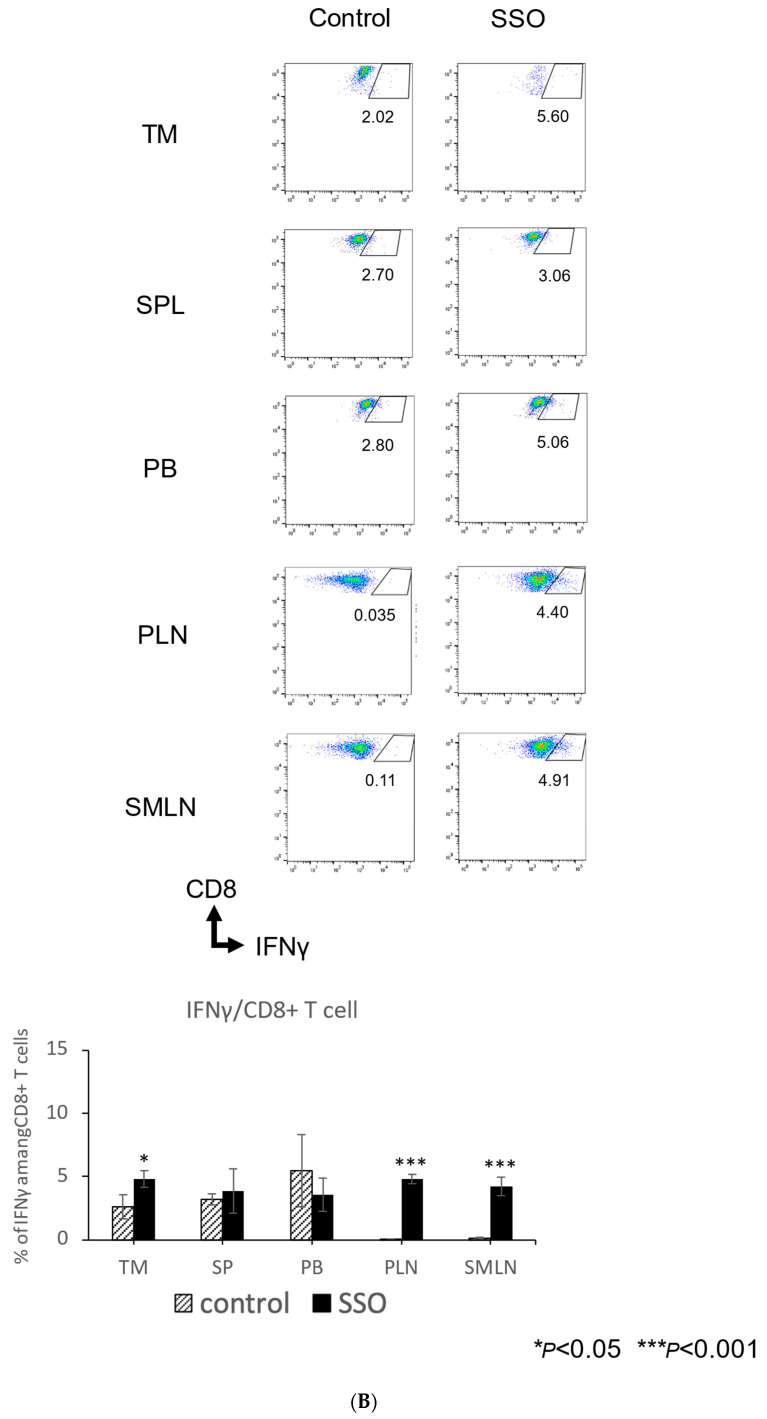

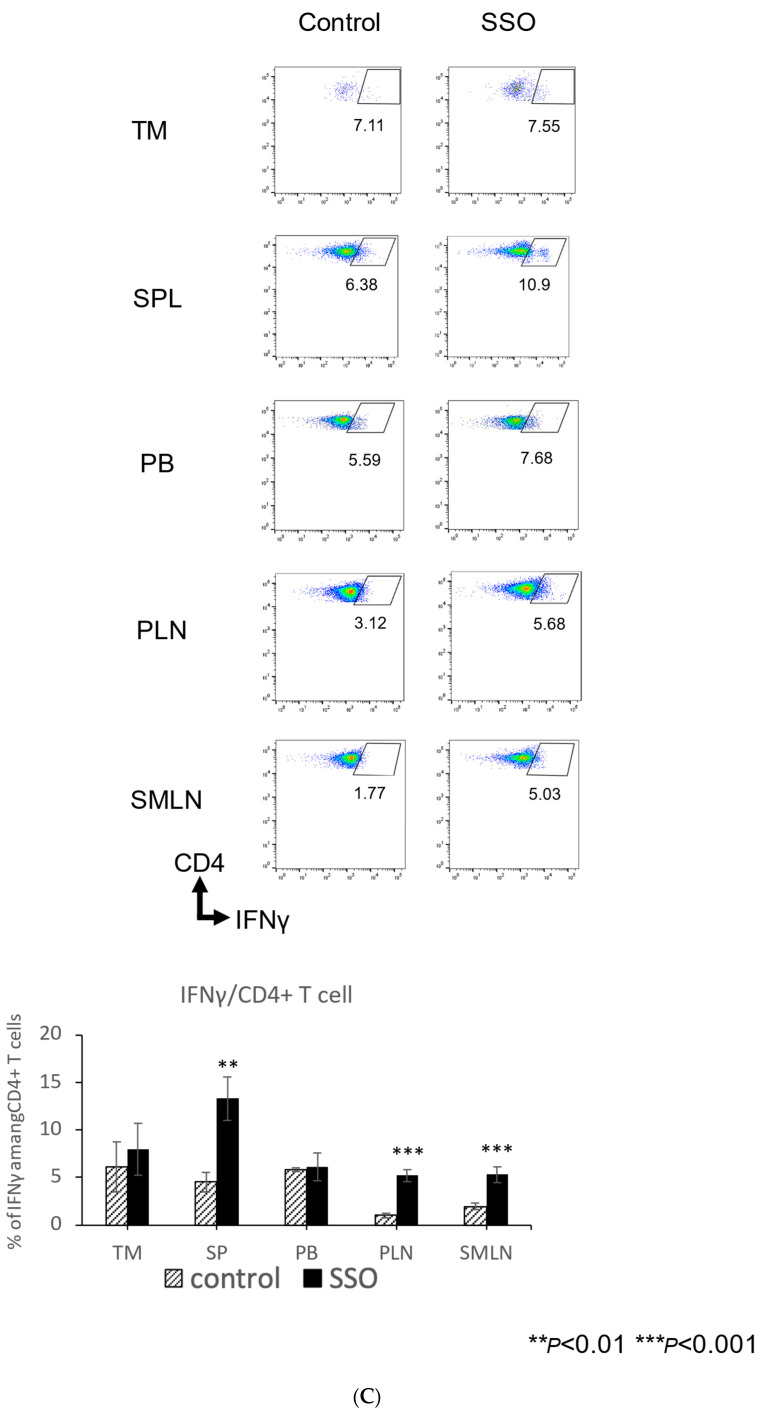

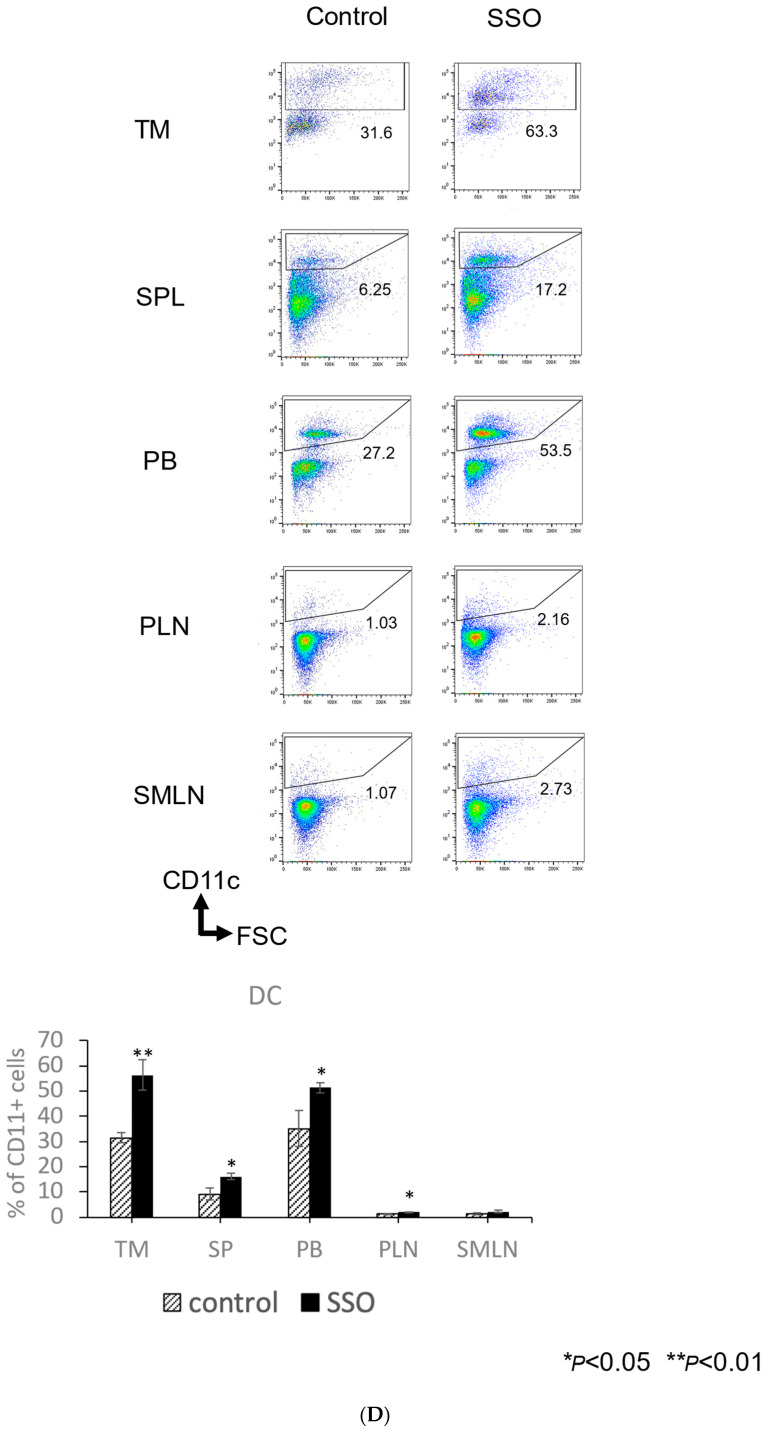

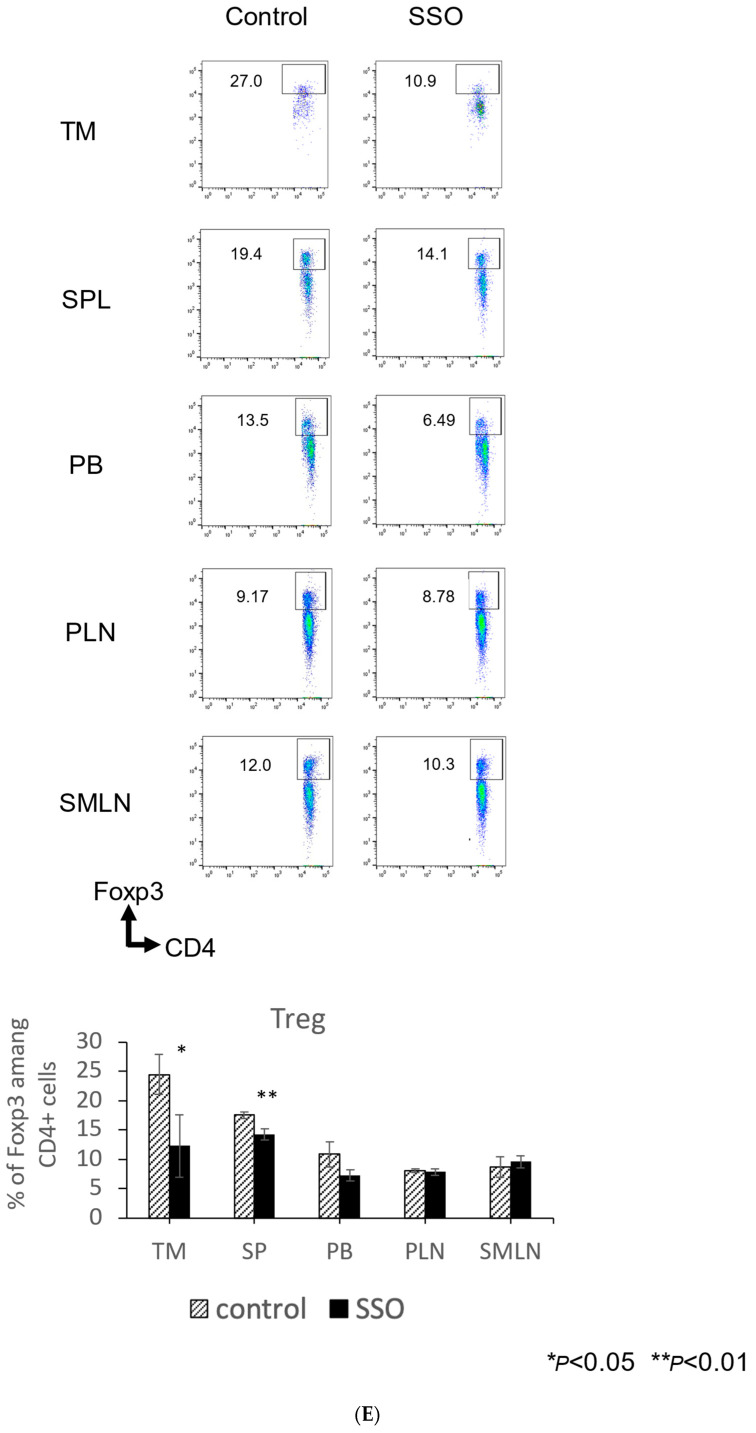

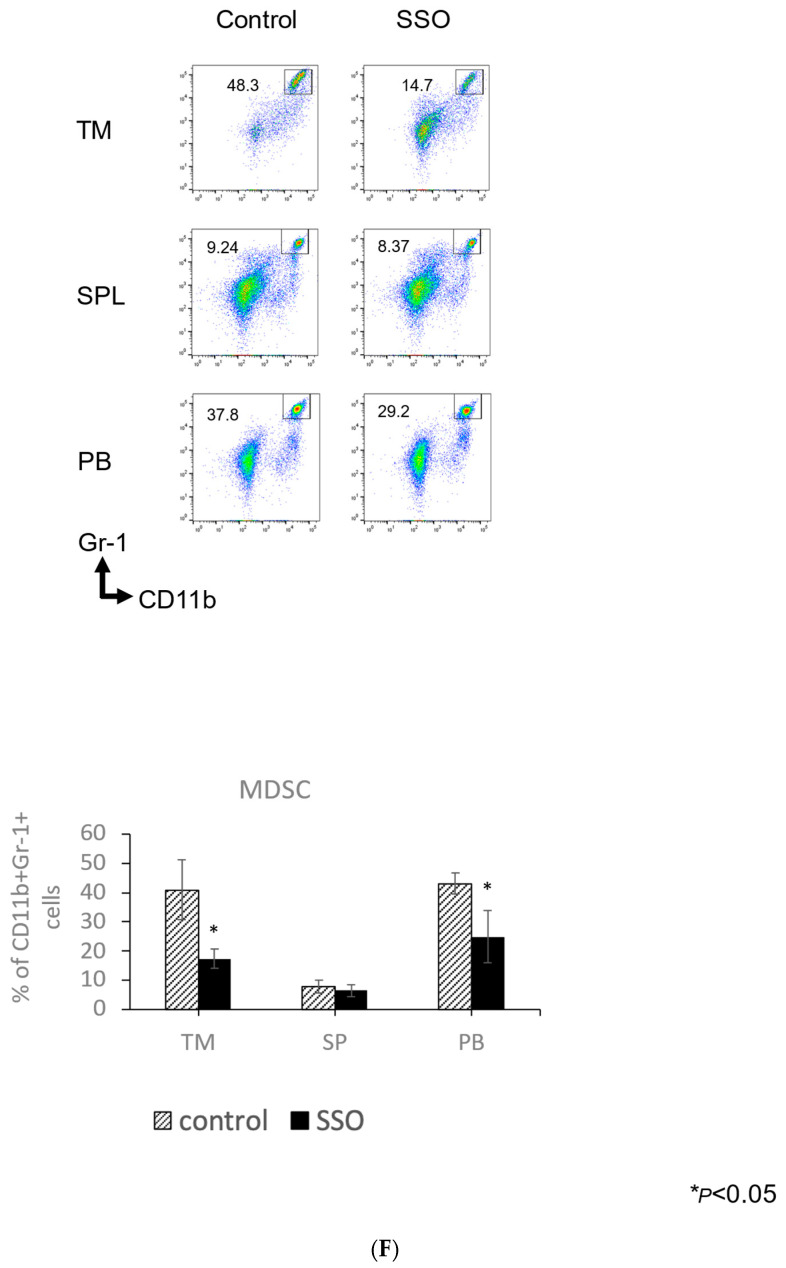
The comparison of the proportions of various immune cells in OSCC-bearing mice between the SSO-treated and non-treated populations. Mice were challenged with NR-S1K cells. The mice were injected intraperitoneally with a dose of 20 mg/kg of SSO every day from day 1 to day 13. After 14 days of tumor inoculation, the tumor, spleen, peripheral blood, peripheral lymph nodes, and submandibular lymph nodes were harvested and the percentages of various immune cells in each organ were determined using flow cytometry: (**A**) CD8^+^ T cells, CD4^+^ T cells, (**B**) IFN-γ-producing CD8^+^ T cells, (**C**) IFN-γ-producing CD4^+^ T cells, (**D**) Dendritic cells (DCs), (**E**) Regulatory T cells (Tregs), and (**F**) Myeloid-derived suppressor cells (MDSCs). Representative scatter plots and a summary of these results are shown (*n* = 3/group); * p < 0.05, ** p < 0.01, *** p < 0.001, NT vs. SSO. (TM: Tumor, SP: spleen, PB: peripheral blood, PLN: peripheral lymph nodes, and SMLN: submandibular lymph nodes).

As shown in Figure 5A, the total proportion of CD8^+^ T cells was significantly increased in the tumors, spleens, peripheral blood, and cervical lymph nodes of the SSO-treated mice compared to the control mice. Furthermore, the total proportion of IFN-γ-producing CD8^+^ T cells was significantly increased in the tumors, peripheral lymph nodes, and cervical lymph nodes of the SSO-treated mice compared to the control mice (Figure 5B). The total proportion of CD4^+^ T cells was significantly increased in the tumors of the SSO-treated mice compared to the control mice (Figure 5A). The total proportion of IFN-γ-producing CD4^+^ T cells was significantly increased in the spleens, peripheral lymph nodes, and cervical lymph nodes of the SSO-treated mice compared to the control mice (Figure 5C). Figure 5D shows that the total proportion of dendritic cells (DCs) was significantly increased in the tumors, spleens, peripheral blood, and peripheral lymph nodes of the SSO-treated mice compared to the control mice. However, as shown in Figure 5E, the total proportion of regulatory T cells (Tregs) was significantly decreased in the tumors and spleens of the SSO-treated mice compared to the control mice. Furthermore, the total proportion of myeloid-derived suppressor cells (MDSCs) was significantly decreased in the tumors and peripheral blood of the SSO-treated mice compared to the control mice (Figure 5F).

These results indicated that the selective inhibition of CD36 could alter the distribution of immune cell populations, which may facilitate antitumor immune responses in oral cancer-bearing hosts.

## 3. Discussion

Recently, the mechanism of lipid metabolism-mediated tumor progression has been elucidated in the TME [8]. It was elucidated that fatty acid synthesis is more abundant in tumor cells compared to normal cells, and fatty acids are used in the proliferation of tumor cells [34,35]. Particularly, fatty acid synthases (FASN) play an important role in the fatty acid-mediated proliferation of tumor cells [35]. It was demonstrated that the selective inhibition of FASN attenuated the progression of prostate cancer, non-small-cell lung cancer, and metastasized breast cancer [36,37,38]. Moreover, the growth-inhibitory effects of cholesterol-lowering drugs have been demonstrated in OSCCs, suggesting a relationship between tumor growth and lipid metabolism [39]. Lipid metabolism is mediated by FA protein transporters in the plasma membrane. Recent studies have revealed that the CD36 expressed by various tumor cells particularly affects tumor growth and metastasis [13,14,15,16,17,18,19,20,21,22,23,24,25].

Furthermore, CD36 is expressed by various immune cells. Particularly, the CD36 expression by immunosuppressive cells such as Tregs and MDSCs in the tumor suggests its contribution to the immunosuppressive function of these cell types in the tumor-bearing hosts [28,29,30,31]. 

Our previous study also revealed that CD36 was abundantly expressed by OSCCs and involved in the proliferation and migration of the tumor cells, whereas little is known about the role of CD36 in the host immune responses in OSCCs [26]. Therefore, we evaluated the role of CD36 in the OSCC host’s immune responses.

In the present study, a CD36 inhibitor was used to target lipid metabolism in OSCCs, and it showed not only direct antitumor effects but also the enhancement of antitumor immune responses, both in vitro and in vivo. Previous studies have revealed that CD36 inhibition enhances T cell immune responses and dendritic cell function in the tumor-bearing hosts of various types of cancers [29,30,31,32,40]. In contrast, the immunosuppressive functions of Tregs and MDSCs were diminished [28,29]. This is due to the energy metabolism of each immune cell: Tregs and MDSCs have enhanced immunosuppressive functions due to enhanced lipid metabolism via CD36 [22,28]. In CD8^+^ T cells, increased fatty acid uptake via CD36 leads to ferroptosis and attenuated effector function [30]. Our results also suggested that CD36 was involved in the regulation of T cell immune responses, dendritic cell functions, and the deregulation of immunosuppressive cell functions in OSCCs, suggesting that lipid metabolism is important in the regulation of antitumour immune responses in OSCCs. Future experiments on the gene modification of CD36 would be desirable, to investigate whether these effects directly attribute to the CD36 inhibition.

In recent years, the emergence of immune ICIs has revolutionized the treatment of recurrent and/or metastatic oral cancer, resulting in a better overall survival rate than conventional therapies [6,7]. However, the response rate of ICIs is limited, and it is expected that combination drugs will be developed to enhance the efficacy of ICIs. It was suggested that one of the causes of poor responses to ICIs is the accumulation of immunosuppressive cells such as Tregs and MDSCs in tumor-bearing hosts, which acquire resistance to ICIs [36,37,38]. Our results and others’ studies suggested that the function of immunosuppressive cells could be decreased by CD36 inhibition, and the effect of ICIs may be increased by their combination with CD36 inhibitors [28,29]. The combination of ICIs and CD36 inhibitor in a hepatocellular carcinoma (HCC) in vivo model significantly improved the growth inhibitory effect and survival rate compared to ICIs alone [41]. Furthermore, antitumor immunomodulatory effects in tumor-bearing hosts have been demonstrated; a decrease in Tregs and MDSCs and an increase in CD8^+^ T cell-producing IFN-γ and granzyme B was shown, suggesting that the combination of ICIs and CD36 inhibitors may have synergistic effects [42]. In the present study, the treatment with CD36 inhibitors of OSCC-bearing mice decreased immunosuppressive cells and increased T cell-producing IFN-γ. Further study would be necessary to elucidate the synergistic effects of ICIs and CD36 inhibitors.

The optimization of combined immunotherapy would require the exploration of therapeutic efficacy and the identification of predictive biomarkers for the therapeutic efficacy simultaneously. CD36 expression is higher in poorly differentiated OSCCs than in well-differentiated OSCCs, and the metastatic ability of CD36-positive OSCCs is higher than in CD36-negative OSCCs, suggesting that CD36 may be an excellent predictive biomarker for the aggressiveness of tumors [22,26]. Moreover, our present study revealed that CD36 inhibition attenuated the immune suppressive properties in OSCC-bearing hosts, suggesting that CD36 may be a potential biomarker for antitumour immune responses in oral cancer. For the development of more personalized medicine for ICI refractory oral cancer patients, further studies are necessary to elucidate the synergistic effects of CD36 inhibitors in combination with ICIs and to evaluate the utility of CD36 expression as a predictive biomarker for the response to immunotherapy.

## 4. Materials and Methods

### 4.1. Mice and Cell Lines

The C3H/HeN mice were purchased from Sankyo Laboratory Services, Inc. All the mice were maintained under specific pathogen-free conditions under the institutional guidelines of the University of Toyama.

The mouse OSCC line NR-S1K was established from the NR-S1 cell line [43]. This cell line was kindly provided by Dr. Masato Azuma, Department of Molecular Immunology, Graduate School, Tokyo Medical and Dental University. The mouse OSCC line SCCVII is an established cell line from the C3H/HeN mouse line [33]. 

### 4.2. Cell Proliferation Assay

Cell proliferation assays were performed, as described previously [43]. The OSCCs were seeded in 96-well plates at a density of 1.5 × 10^4^ cells/well and cultured for 72 h in the presence or absence of various concentrations of SSO. The viability of adherent cells was measured by adding (WST-1) premix (Takara Bio, Kusatsu, Japan), according to the manufacturer’s instructions.

### 4.3. Assessment of Cellular Apoptosis

The OSCCs were seeded in 6-well plates at a density of 1.0 × 10^5^ cells/well and cultured for 48 h in the presence or absence of 50 μM SSO. Apoptotic cell death was detected using Annexin V (BD Pharmingen, San Diego, CA, USA) staining, according to the manufacturer’s instructions, as described previously [44]. 

### 4.4. Assessment of Phenotypic Alteration of Cells

The OSCCs were seeded in 6-well plates at a density of 1.0 × 10^5^ cells/well and cultured in the presence or absence of 50 μM SSO for 48 h. Cells were collected and stained with antibodies against each cell surface molecule. The samples were analyzed by flow cytometry.

### 4.5. Mixed Lymphocyte Reaction (MLR) and Intracellular Cytokine Staining

Mixed Lymphocyte Reaction (MLR) assays were performed, as previously described [45]. The OSCCs were seeded in 6-well plates at a density of 1.0 × 10^5^ cells/well and cultured in the presence or absence of 50 μM SSO for 72 h. The total spleen cells from naive mice and the OSCCs were cocultured for 72 h in the presence of 1 μg/mL anti-CD3 antibodies (spleen cells:OSCCs = 10:1).

### 4.6. In Vivo SSO Treatment

The NR-S1K cells (2.0 × 10^6^) were administered subcutaneously into the right masseter of C3H/HeN mice. The mice were injected intraperitoneally with 20 mg/kg SSO every day from day 1 to day 13. The SSO was dissolved in a minimal amount of DMSO and then dissolved in the saline for in vivo administration. The same amount of saline was administered to the control mice. After 14 days of tumor inoculation, mice were humanely sacrificed, and the tumor, peripheral blood, lymph nodes, and spleen were extracted, and the cells were analyzed by flow cytometry. The tumor size (length × width) was measured daily from the first i.p. injection to monitor the tumor’s size progression.

### 4.7. Antibodies and Reagents

The following antibodies were purchased from eBioscience (San Diego, CA, USA): FITC-conjugated anti-mouse CD40, CD54(I-CAM), CD83, CD273(B7-DC, PD-L2); PE-conjugated anti-mouse CD80, MHC Class, PD-L1, CD62P(P-Selectin); PerCP-Cy5.5-conjugated anti-mouse; PE-Cy7-conjugated anti-mouse IFN-γ, CD8, CD19; APC-conjugated anti-mouse CD106(VCAM-1), CD8, Foxp3; and APC-Cy7 conjugated anti-mouse Ly-6G/Ly-6C(Gr-1). The following antibodies were purchased from TONBO biosciences (Osaka, Japan): PE-conjugated anti-mouse CD11c and APC-conjugated anti-mouse CD86, MHC class II. The following antibodies were purchased from Biolegend (San Diego, CA, USA)**:** FITC-conjugated anti-mouse CD11b; PE-conjugated anti-mouse IFN-γ; PerCP-Cy5.5-conjugated anti-mouse CD4, CD45R(B220); APC-Cy7 conjugated anti-mouse CD3; and BV510-conjugated anti-mouse CD45. Carboxyfluorescein diacetate succinimidyl ester (CFSE) was purchased from Invitrogen (Carlsbad, CA, USA). Sulfosuccinimidyl oleate sodium (SSO) was purchased from Abcam (Cambridge, UK).

### 4.8. Flow Cytometry

A flow cytometry analysis was performed as described previously [45]. Samples were assessed using FACS Celesta (Becton Dickinson, San Jose, CA, USA).

### 4.9. Statistical Analysis

Group comparisons were made using Student’s *t*-test or ANOVA with the statistical software OriginPro 2018 (OriginLab Corporation, Northampton, MA, USA), and a value of *p* < 0.05 was considered statistically significant.

## 5. Conclusions

Overall, our data suggest that CD36 is indispensable in the proliferation and survival of oral cancer cells and regulates various immunological functions in oral tumor-bearing hosts. Because the synergistic effect of CD36 inhibitors and immunotherapy has generated much interest as a new therapeutic strategy, CD36 may be a potential therapeutic target for cancer therapy as a central regulator for tumor progression and immune evasion in tumor-bearing hosts. Further studies are necessary to elucidate an effective and safe clinical setting for CD36 inhibitors and to develop a more personalized medicine for oral cancer treatment. 

## Data Availability

Any personal or patient data are unavailable due to privacy or ethical restrictions. All other data are available from the corresponding author upon reasonable request.

## Supplementary Materials

The following supporting information can be downloaded at: https://www.mdpi.com/article/10.3390/ijms25179438/s1.
