# Supplementary material for: Targeting CD36-Mediated Lipid Metabolism by Selective Inhibitor-Augmented Antitumor Immune Responses in Oral Cancer
Source: Int J Mol Sci. 2024 Aug 30;25(17):9438. doi: 10.3390/ijms25179438 (PMC11395596; doi:10.3390/ijms25179438)
Supplement: Supplementary file 1 [file ijms-25-09438-s001.zip › ijms-3116501 - supplmentary figure s1.pdf]

Supplement Figure. 1

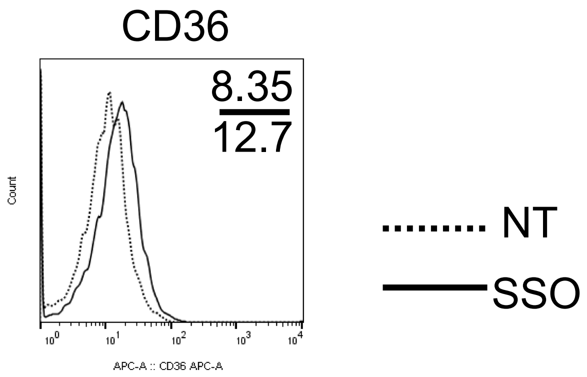

Supplement Figure 1. Phenotypic alteration on OSCC cells by SSO. OSCC cells were cultured in the presence or absence of 50μM SSO for 48 hours, and the cell surface expression of CD36 was analyzed using flow cytometry. Experiments were performed in triplicate and similar results were obtained. Representative histograms from one experiment are shown. Dashed lines and solid lines indicate results for non-treated (NT) cells and SSO-treated cells, respectively. Numbers in each panel indicate the mean fluorescence intensity of each molecule in NT cells (upper) and SSO-treated cells (lower).
